# Liver Fibrosis in HCV Monoinfected and HIV/HCV Coinfected Patients: Dysregulation of Matrix Metalloproteinases (MMPs) and Their Tissue Inhibitors TIMPs and Effect of HCV Protease Inhibitors

**DOI:** 10.3390/ijms17040455

**Published:** 2016-03-26

**Authors:** Tiziana Latronico, Claudia Mascia, Ilaria Pati, Paola Zuccala, Fabio Mengoni, Raffaella Marocco, Tiziana Tieghi, Valeria Belvisi, Miriam Lichtner, Vincenzo Vullo, Claudio Maria Mastroianni, Grazia Maria Liuzzi

**Affiliations:** 1 Department of Biosciences, Biotechnologies and Biopharmaceutics, Aldo Moro University, Bari 70126, Italy; tiziana.latronico@uniba.it (T.L.); ilaria.pati@hotmail.it (I.P.); graziamaria.liuzzi@uniba.it (G.M.L.); 2 Department of Public Health and Infectious Diseases, Sapienza University, Rome 00185, Italy; claumascia@tiscali.it (C.M.); paola.zuccala@libero.it (P.Z.); fabio.mengoni@uniroma1.it (F.M.); tizianatieghi@libero.it (T.T.); valeria.belvisi@gmail.com (V.B.); miriam.lichtner@uniroma1.it (M.L.); vincenzo.vullo@uniroma1.it (V.V.); 3 Infectious Diseases Unit, Sapienza University, Polo Pontino, Latina 04100, Italy; raffa.marocco@libero.it

**Keywords:** matrix metalloproteinases, tissue inhibitors of metalloproteinases, HIV/HCV coinfection, liver fibrosis, anti-HCV therapy

## Abstract

An imbalance between matrix metalloproteinases (MMPs) and tissue inhibitors of metalloproteinases (TIMPs) may contribute to liver fibrosis in patients with hepatitis C (HCV) infection. We measured the circulating levels of different MMPs and TIMPs in HCV monoinfected and HIV/HCV coinfected patients and evaluated the potential for anti-HCV therapy to modulate MMP and TIMP levels in HCV subjects. We analyzed 83 plasma samples from 16 HCV monoinfected patients undergoing dual or triple anti-HCV therapy, 15 HIV/HCV coinfected patients with undetectable HIV load, and 10 healthy donors (HD). Levels of MMP-1, MMP-2, MMP-3, MMP-8, MMP-9, MMP-10, TIMP-1, and TIMP-2 were measured by a SearchLight Multiplex Immunoassay Kit. MMP-2 and MMP-9 were the highest expressed MMPs among all the analyzed samples and their levels significantly increased in HCV monoinfected and HIV/HCV coinfected subjects compared to HD. TIMP-1 levels were significantly higher in HCV and HIV/HCV subjects compared to HD and were correlated with liver stiffness. These findings raise the possibility of using circulating TIMP-1 as a non-invasive marker of liver fibrosis in HCV infection. A longitudinal study demonstrated that MMP-9 levels significantly decreased (40% reduction from baseline) in patients receiving dual as well as triple direct-acting antivirals (DAA) anti-HCV therapy, which had no effect on MMP-2, TIMP-1, and TIMP-2. As the dysregulation of MMP-2 and MMP-9 may reflect inflammatory processes in the liver, the decrease of MMP-9 following HCV protease inhibitor treatment suggests a positive effect on the reduction of liver inflammation.

## 1. Introduction

Chronic infection with hepatitis C virus (HCV) is characterized by progressive damage to liver tissue leading to progressive fibrosis and evolving ultimately to cirrhosis, liver failure, and hepatocellular carcinoma [1]. Liver fibrosis is a dynamic pathologic process characterized by an accumulation of the extracellular matrix, which is a consequence of an imbalance between the enhanced matrix synthesis and reduced breakdown of connective tissue proteins reflecting dysregulation of several pathways, including matrix metalloproteinases (MMPs) and their natural tissue inhibitors (TIMPs) [2] MMPs are a large family of zinc-dependent endopeptidases that degrade the components of extracellular matrix and basement membrane [3]. The activity of MMPs is highly regulated both at the level of gene expression and by activation of latent pro-MMPs to active enzymes [4]. In the extracellular milieu, the activity of these enzymes is controlled by TIMPs, which form stable, non-covalent complexes with active MMPs [5]. Changes in the fine balance between MMPs and TIMPs drives the turnover of the extracellular matrix and may be crucial for inflammation in infection as well as other pathological conditions. With regard to HCV, several data in literature indicated that hepatic fibroproliferation has been associated with alterations in the expression of circulating and hepatic MMPs and TIMPs [6,7,8], but results obtained are controversial and the information is fragmentary as most studies are limited to the analysis of individual MMPs and/or TIMPs.

In recent years HCV has emerged as an important opportunistic pathogen among patients with HIV diseases [9] and infection with HIV appears to adversely affect the outcome of chronic HCV hepatitis. A synergy between the two viruses leads to a higher rate of viral persistence, an increase of viral load, higher rates of hepatic decompensation, and an increase in the likelihood of oncogenesis with a faster rate of liver fibrosis progression [10]. Retrospective studies reported that coinfected patients were more likely to develop severe liver fibrosis and cirrhosis [11,12], but the reasons for the accelerated progression of hepatic fibrosis in HIV/HCV coinfected patients are not well elucidated.

Hence, advances in the understanding of the pathophysiology and regulation of fibrosis are fundamental for the development of therapeutic strategies. Different data suggest that HIV suppression achieved with antiretroviral therapy (ART) ameliorates HCV-related liver disease progression and reduces liver-related mortality in HIV/HCV coinfected patients [13].

We have previously demonstrated that HIV/HCV coinfected subjects exhibit an altered balance between circulating MMP-9 and TIMP-1 [14], probably due to the chronic state of immune activation and cytokine dysregulation associated with HIV disease [15]. Although the effects of antiretroviral therapy have been studied on some MMPs and TIMPs in the course of HIV/HCV coinfection, little is known about the effect of antivirals in the course of HCV infection.

The current anti-HCV therapies are not designed to be anti-fibrotic but are focused on virus eradication. For many years, treatment of chronic HCV has been inadequate. So far the standard therapy of chronic HCV infection is based on a combination of pegylated interferon-α (peg-IFNα) and ribavirin (RBV). Although this combination can lead to a sustained virologic response (SVR), it does not prevent future reinfection and is less efficacious in patients with advanced disease [16]. The introduction of direct-acting antiviral drugs (DAAs)—with the two first-generation protease inhibitors (PI), boceprevir and telaprevir—has increased the number of patients who respond to treatment [17]. In the present study, we examined a panel of MMPs and TIMPs in plasma samples from HCV monoinfected patients and HIV/HCV coinfected patients under effective antiretroviral therapy. We also assessed the dynamic changes of MMPs and TIMPs in HCV monoinfected patients receiving dual therapy with peg-IFNα and RBV (PR) or triple anti-HCV therapy with PR plus first-generation HCV NS3/4A protease inhibitors (telaprevir or boceprevir).

## 2. Results

### 2.1. Detection of Different Matrix Metalloproteinase (MMP) and Tissue Inhibitors of Metalloproteinase (TIMP) Levels in Plasma of Hepatitis C Virus (HCV) Monoinfected, HIV/HCV Coinfected Subjects, and Healthy Donors

As reported in Table 1, the immunoassay analysis of plasma from healthy donors (HD), coinfected HIV/HCV and monoinfected HCV subjects, at time of recruitment, indicated that, with the exception of MMP-13, all the analyzed MMPs and TIMPs were detectable in the samples of the analyzed subject categories. In particular, levels of MMP-1, MMP-2, MMP-9, TIMP-1, and TIMP-2 were significantly higher in samples from HCV and HIV/HCV patients in comparison with HD.

### 2.2. Plasma Levels of MMP-2, MMP-9, TIMP-1, and TIMP-2 in the Different Groups Analyzed

**Figure 1:**
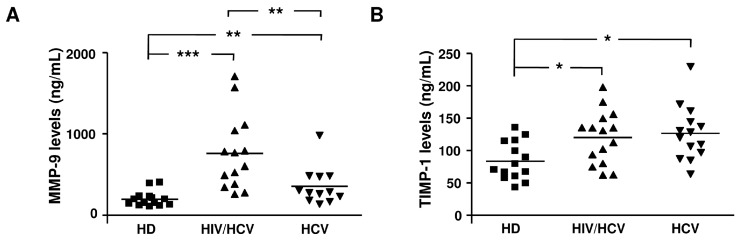

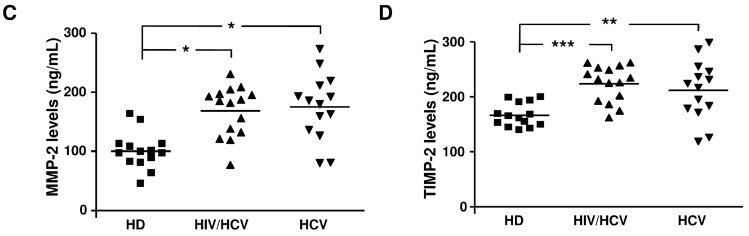
Distribution of MMP-2 (MMP, matrix metalloproteinase), MMP-9, TIMP-1 (TIMP, tissue inhibitors of metalloproteinase), and TIMP-2 levels in plasma from healthy donors, HIV/HCV coinfected, and HCV monoinfected subjects. MMP-9 (**A**), TIMP-1 (**B**), MMP-2 (**C**), and TIMP-2 (**D**) were detected at baseline in plasma from healthy donors (HD), HIV/HCV coinfected, and HCV monoinfected subjects. The quantification of MMPs and TIMPs in the samples was determined using a calibration curve obtained by known concentrations of MMPs and TIMPs standard. The reported scatter plots show the distribution of MMP-2, MMP-9, TIMP-1, and TIMP-2 levels, expressed as ng/mL, in the plasma of the different analyzed categories. Horizontal bars indicate the median and asterisks represent values statistically different among the analyzed categories (* *p* < 0.05; ** *p* < 0.01; *** *p* < 0.001) (one-way Anova followed by the *post hoc* Tukey’s Multiple Comparison Test).

Figure 1 shows the distribution of MMP-2 and MMP-9, which were the highest expressed MMPs, as well as of TIMP-1 and TIMP-2, in plasma samples from subjects of the different categories analyzed. A statistically significant increase of MMP-2, MMP-9, TIMP-1, and TIMP-2 levels was detected in HCV monoinfected and HIV/HCV coinfected subjects compared to HD. Levels of MMP-2, TIMP-1, and TIMP-2 were comparable in HCV and HIV/HCV subjects, whereas a statistically significant increase of MMP-9 levels was found in HIV/HCV coinfected subjects in comparison with HCV monoinfected subjects.

### 2.3. Correlation between Liver Stiffness and TIMP-1 Levels in HCV Monoinfected and HIV/HCV Coinfected Subjects

**Figure 2:**
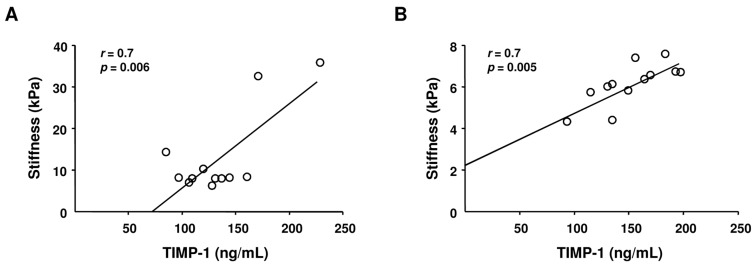
Correlation between liver stiffness and TIMP-1 levels in HCV monoinfected (**A**) and HIV/HCV coinfected (**B**) subjects. According to Pearson’s correlation coefficient, a statistically significant positive correlation was observed between liver stiffness (kPa) and TIMP-1 levels (ng/mL). *r* and *p* Pearson correlation coefficients are indicated.

As shown in Figure 2, a statistically significant correlation was found in HCV monoinfected (Figure 2A) and HIV/HCV coinfected subjects (Figure 2B) between TIMP-1 plasma levels and liver stiffness at time of recruitment, indicating the existence of a relationship between TIMP-1 and liver fibrosis. The receiver operating characteristic (ROC) curve of plasma TIMP-1 levels, which represents the relationship between the sensitivity and the specificity of plasma TIMP-1 in detecting liver fibrosis, showed that a cutoff value of 89 ng/mL resulted in 91.67% sensitivity and 83.33% specificity for HCV monoinfected and 100% sensitivity and 84.43% specificity for HIV/HVC coinfected patients

### 2.4. TIMP-1/MMP-9 Ratio between HCV Monoinfected and HIV/HCV Coinfected Subjects

**Figure 3:**
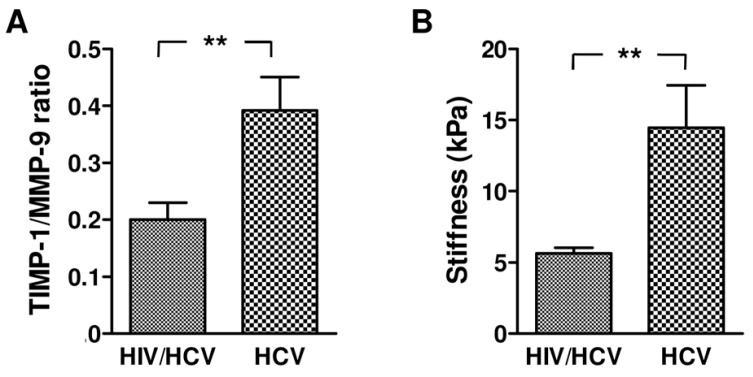
TIMP-1/MMP-9 ratio and liver stiffness in HCV monoinfected and HIV/HCV coinfected subjects. The histograms in Figure 3**A** represent the ratio between TIMP-1 and MMP-9 levels detected at baseline (T0) in plasma from HIV/HCV coinfected and HCV monoinfected subjects; The histograms in Figure 3**B** represent the degree of liver stiffness calculated by ultrasound based transient elastography using FibroScan. Asterisks represent values statistically different among the analyzed categories (** *p* < 0.01) (Student’s *t*-test).

We also calculated the enzyme/inhibitor ratio, which provides a more comprehensive way to assess the net activity of MMPs. As shown in Figure 3A a higher TIMP-1/MMP-9 ratio was observed in HCV monoinfected subjects in comparison with HIV/HCV coinfected subjects. These findings are consistent with the degree of liver fibrosis, which in our study population was more severe in HCV monoinfected patients when compared with HIV/HCV coinfected patients (Figure 3B).

### 2.5. Effect of Dual and Triple Anti-HCV Therapy on MMPs and TIMPs in HCV Monoinfected Subjects

**Figure 4:**
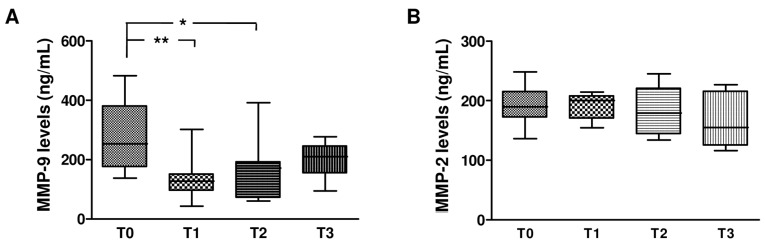

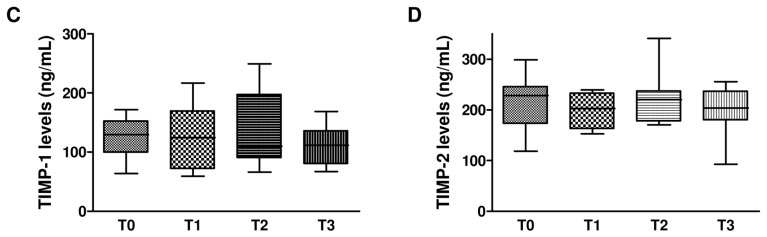
Longitudinal evaluation of MMP-2, MMP-9, TIMP-1 and TIMP-2 levels in plasma from HCV monoinfected individuals subjected to dual therapy. The box plots in Figure 4 represent levels (ng/mL) of MMP-9 (**A**), MMP-2 (**B**), TIMP-1 (**C**), and TIMP-2 (**D**) in plasma from HCV monoinfected subjects at the time of recruitment (T0, baseline) and after 3 (T1), 6 (T2), and 12 (T3) months of treatment with dual anti-HCV therapy with pegylated interferon-α (peg-IFNα) and ribavirin (RBV). Horizontal bars indicate the medians. Asterisks represent values statistically different from baseline (* *p* < 0.05; ** *p* < 0.01) (one-way Anova followed by the *post hoc* Tukey’s Multiple Comparison Test).

**Figure 5:**
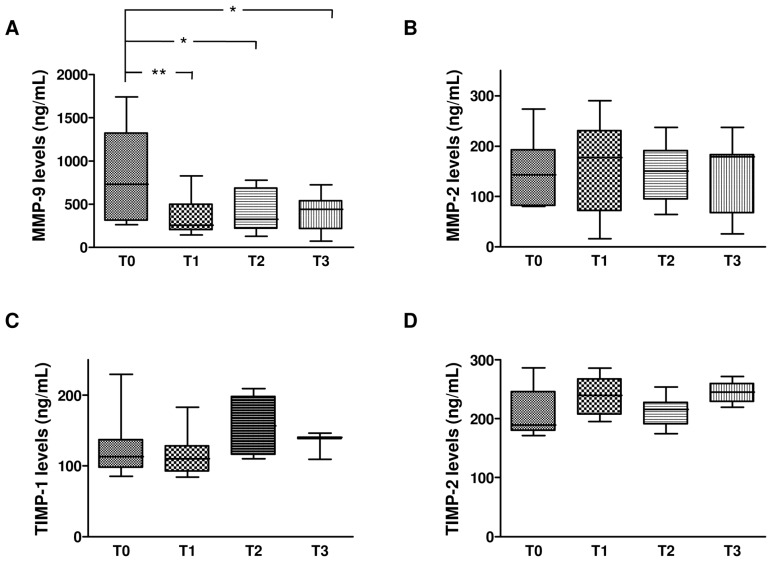
Longitudinal study on the effect of triple therapy on MMP-2, MMP-9, TIMP-1, and TIMP-2 in plasma from HCV monoinfected subjects. The box plots in Figure 5 represent levels (ng/mL) of MMP-9 (**A**), MMP-2 (**B**), TIMP-1 (**C**) and TIMP-2 (**D**) in plasma from HCV monoinfected subjects at the time of recruitment (T0, baseline) and after 3 (T1), 6 (T2), and 12 (T3) months of treatment with triple anti-HCV therapy with pegylated interferon-α (peg-IFNα) and ribavirin (RBV) + HCV NS3/4A protease inhibitors (telaprevir or boceprevir). Horizontal bars indicate the medians. Asterisks represent values statistically different from baseline (* *p* < 0.05; ** *p* < 0.01) (one-way Anova followed by the *post hoc* Tukey’s Multiple Comparison Test).

We monitored for 12 months the changes of MMP-2, MMP-9, TIMP-1, and TIMP-2 levels in plasma samples from HCV monoinfected subjects treated with dual (Figure 4) or triple therapy (Figure 5). In particular, the plasma levels of MMPs and TIMPs were assessed in the HCV-infected patients at time of recruitment (T0, baseline) and after 1 (T1), 6 (T2), and 12 (T3) months of treatment.

As shown in Figure 4 and Figure 5, at baseline no differences were detected in the levels of MMP-2, TIMP-1, and TIMP-2 between subjects treated with dual or triple therapy. In contrast, MMP-9 baseline levels were highest in the subjects that had been treated with triple therapy in comparison with those subjected to dual therapy. However, the longitudinal study highlighted that both therapies reduced MMP-9 levels (40% reduction) in comparison with their respective baseline levels, whereas they had no effect on MMP-2, TIMP-1, and TIMP-2. In particular, a significant decrease of MMP-9 levels was reported in the first month of dual anti-HCV therapy, followed by a gradual increase until the 12th month. Conversely, triple anti-HCV therapy induced a statistically significant reduction of MMP-9 levels that remained constant during the entire treatment period.

## 3. Discussion

Although dysregulation of MMPs and TIMPs has been observed in the course of HCV infection [18,19,20], a more detailed analysis of the pattern of these enzymes and their inhibitors is needed to clarify the role of MMPs and TIMPs in the development of liver fibrosis in the course of HCV monoinfection and HIV/HCV coinfection.

In this study, we analyzed an extensive panel of circulating MMPs and observed an increase of plasma levels of MMP-1 (collagenase-1), MMP-2 (gelatinase A), and MMP-9 (gelatinase B) in HCV and HIV/HCV subjects in comparison to HD. Among the studied MMPs we focused our attention on MMP-2 and MMP-9 for two different reasons: (i) they were the highest expressed MMPs among those analyzed; (ii) in the family of MMPs, MMP-2 and MMP-9 are particularly important for the development of liver fibrosis since they degrade type IV collagen (basal membrane) and therefore are involved in the early steps of tissue remodeling that characterizes chronic liver disease [21,22]. In this study, we found that MMP-9 plasma concentrations significantly increased in HIV/HCV coinfected subjects compared to HCV monoinfected subjects, despite the fact that HIV/HCV coinfected subjects were treated with antiretroviral therapies, which are known to inhibit MMP-9 levels [23,24]. To explain this result, we suggest that in HIV/HCV coinfected subjects antiretroviral therapy is not able to counteract the persistent chronic inflammatory state amplified by the synergic effect of the two viruses.

In this study we also found an increase of TIMP-1 and TIMP-2 levels in both HIV/HCV coinfected and HCV monoinfected subjects, without significant differences between the two groups. In HCV monoinfected as well as in HIV/HCV coinfected patients, plasma TIMP-1 levels were correlated with stiffness and discriminated between patients with and without liver fibrosis with both high sensitivity and specificity, raising the possibility of using circulating TIMP-1 as a non-invasive marker of liver fibrosis not only in HCV infection [18,25] but also in HIV/HCV coinfection [26]. The balance between MMPs and TIMPs is a key regulatory factor *in vivo* since it allows us to determine the net activity of MMPs and regulate their action in both pathological and physiological conditions. MMPs are known to degrade the components of the extracellular matrix (ECM), therefore an increased production of MMPs has been implicated in tissue damage. By contrast, an excess of TIMP production may be associated with ECM apposition. Therefore, it is important to determine not only the levels of the isolated enzymes or inhibitors, but also the ratio of enzyme to inhibitor. In our experiments we found a significant increase in the ratio TIMP-1/MMP-9 in HCV monoinfected in comparison with HIV/HCV coinfected subjects. This imbalance toward TIMP-1 may justify the highest levels of stiffness in HCV monoinfected individuals, suggesting that the TIMP-1/MMP-9 ratio mirrors the progression of hepatic fibrosis better than TIMP-1 levels.

In our study we found a lower degree of liver stiffness in our population of HIV/HCV coinfected patients in comparison to the HCV monoinfected subjects. Before the advent of ART, cirrhosis progression was more frequent in HIV/HCV coinfection than in HCV monoinfection [27,28]. In addition, fibrosis progression and the clinical progression to liver decompensation or death were shown to be accelerated in HIV/HCV coinfected patients [29] with advanced immunodeficiency [30]. By contrast, slower fibrosis progression [31] and reduced liver-related mortality were observed in HIV/HCV coinfected patients treated with ART [32]. In addition, a recent study showed a reduction in liver fibrosis scores in HIV-coinfected subjects after 48 weeks of ART treatment [33]. Therefore, although other factors such as heavy alcohol consumption or the duration of HCV infection may be associated with the progression of liver fibrosis, we cannot exclude the possibility that the lower degree of liver stiffness found in our population of HIV/HCV coinfected patients could be due to the effect of ART treatment.

Until 2011, the standard of care for management of chronic HCV hepatitis was the combination of dual therapy with pegylated interferon-α plus ribavirin (PR). More recently, the landscape for the therapy of HCV infection has dramatically changed with the advent of direct-acting antiviral agents (DAAs) [17].

In the present study, we longitudinally assessed the levels of MMPs and TIMPs in HCV monoinfected patients receiving dual PR therapy or triple therapy including PR + first generation HCV protease inhibitors telaprevir or boceprevir. A rapid decline of MMP-9 levels was already evident in the first month of both treatments, suggesting that such therapies may mediate the tissue remodeling and liver inflammation associated with HCV-infection, through their effect on MMP-9 levels. However, the triple therapy was more effective since the MMP-9 levels remained persistently low until the end of treatment. Therefore, the benefits yielded by the HCV protease inhibitors may be ascribed not only to the suppression of virus replication but also to other extravirologic effects. In previous studies, we demonstrated that antiretroviral protease inhibitors have the capability to directly inhibit MMP-2 and MMP-9 secretion and expression in lipopolysaccharide (LPS)-activated glial cells [23]. In addition, HIV protease inhibitors are able to significantly downregulate the expression of MMP-9 in mononuclear cells from HIV-infected individuals [24]. Based on these findings, we can hypothesize that HCV protease inhibitors could also downregulate MMPs with mechanisms that are independent from their capability to block HCV replication. Future studies with newer interferon-free and ribavirin-free regimens could give more information on the dynamic changes of MMPs and TIMPs during treatment and their correlation with virologic response to treatment also in HIV/HCV coinfected patients.

## 4. Materials and Methods

### 4.1. Study Design

We assessed 83 plasma samples from 16 HCV monoinfected subjects and 15 HIV/HCV coinfected subjects recruited from the Infectious Diseases Unit, Sapienza University, Latina, Italy. The HCV-infected patients, at time of recruitment, had never been treated with anti-HCV drugs and started therapy when they were enrolled in the study, after the first collection of blood. Of these patients, eight were treated with dual anti-HCV therapy with pegylated interferon-α (peg-IFNα) and ribavirin (RBV) (PR), whereas eight were treated with triple therapy including PR + HCV NS3/4A protease inhibitors telaprevir or boceprevir. HCV-treated patients were monitored after 1 (T1), 6 (T2), and 12 (T3) months from the beginning of the therapy. The degree of liver stiffness was calculated by ultrasound-based transient elastography using FibroScan. The 15 subjects coinfected with HCV and HIV were receiving antiretroviral therapy and had plasma HIV-RNA plasma levels <20 copies/mL. The control group included 14 healthy donors (HD) who did not show symptoms of viral infection at least 15 days before and after blood donation. The demographic and clinical characteristics of HIV/HCV and HCV subjects and HD as well as the biochemical parameters at baseline are reported in Table 2.

### 4.2. Detection of MMP and TIMP Levels in Plasma Samples

The circulating plasma levels of MMP-1, MMP-2, MMP-3, MMP-8, MMP-9, MMP-10, MMP-13, TIMP-1, and TIMP-2 were assessed by the SearchLight Multiplex Immunoassay Kits (Aushon BioSystems, Billerica, MA, USA) according to the manufacturer’s instructions. This Immunoassay Kit is a multiplex antibody array that measures multiple proteins simultaneously within a single sample increasing sensitivity over the uniplex enzyme immunoassays like Elisa. For the analysis the plasma was diluted 1:100 with the provided diluent. The quantification of MMPs and TIMPs in the samples was determined using a calibration curve obtained using known concentrations of standards.

### 4.3. Statistical Analysis

Parametric one-way analysis of variance, ANOVA, or the non-parametric Kruskal–Wallis test were used to compare the three groups of healthy controls (HD), HCV monoinfected, and HIV/HCV coinfected subjects, as appropriate. In cases of statistical significance, the parametric Tukey’s Multiple Comparison *post hoc* test or the non-parametric Mann–Whitney test was used for the pair-wise comparison of groups. The χ-Square was used for comparison of gender percentages. Student’s T-test was used to compare TIMP-1/MMP-9 ratio and liver stiffness in HCV monoinfected and HIV/HCV coinfected subjects.

The correlation between liver stiffness and TIMP-1 levels in HCV monoinfected and HIV/HCV coinfected subjects was performed using the Pearson’s correlation coefficient. Reported P values were considered statistically significant at *p* < 0.05.

Data were analyzed by the GraphPad Prism 5.0 (GraphPad Software, San Diego, CA, USA).

## Figures and Tables

**Table 1:** Matrix metalloproteinase (MMP) and tissue inhibitors of metalloproteinase (TIMP) levels in plasma samples from healthy donors (HD), plasma of hepatitis C virus (HCV) monoinfected and HIV/HCV coinfected subjects at baseline.

Enzyme or Inhibitor	HD	HCV/HIV	HCV
MMP-1 (ng/mL)	3.5 (0.2–11)	26 (7.6–56) ***	20.5 (0.8–37) ***
MMP-2 (ng/mL)	96.7 (54–163.4)	185 (77–231) **	183 (80–274) **
MMP-3 (ng/mL)	1 (1–3.5)	1 (3.9–1.14)	1 (1–20)
MMP-8 (ng/mL)	8.3 (1.5–66)	15.3 (4.6–147)	18 (1.6–49.4)
MMP-9 (ng/mL)	171 (107–403)	685 (260–1707) ***	295 (137–1000) *
MMP-10 (ng/mL)	0.02 (0.2–2.9)	1.1 (0.02–8)	1.1 (0.02–4.4)
TIMP-1 (ng/mL)	79 (43–135)	130.6 (62–198) *	124 (63.3–230) *
TIMP-2 (ng/mL)	161 (139–200)	232 (162–262) ***	220 (119–300) **

Data represent the median and the range values. Values significantly different in comparison to HD are indicated by asterisks (one-way ANOVA followed by Tukey’s Multiple Comparison Test; * *p* < 0.05, ** *p* < 0.01, and *** *p* < 0.001).

**Table 2:** Demographic and clinical parameters of healthy donors (HD), HCV monoinfected, and HIV/HCV coinfected subjects.

Parameters	HD (*n* = 12)	HCV (*n* = 16)	HIV/HCV (*n* = 15)	*p* Value
Age, Years	46 (28–56)	46 (24−66)	53 (47−57)	0.02 ^a^
Male/Female (% male)	3/9 (25%)	11/5 (68%)	7/8 (46%)	0.07 ^b^
Liver Stiffness (kPa)	–	10 (6.1−35.8)	6.1 (4.3−29.5)	0.03 ^c^
ALT (UI/L)	–	98 (40−450)	58 (24−115)	0.09 ^c^
AST (UI/L)	–	65.5 (27−141)	43 (24−79)	0.24 ^c^
GGT (UI/L)	–	48.5 (17−134)	44 (20−665)	0.9 ^c^
PLT (10^9^/L)	–	168 (89−385)	167 (89−332)	0.06 ^c^
Tot BIL (mg/dL)	–	0.64 (0.38−1.12)	0.8 (0.39−4.3)	0.39 ^c^
HCV-RNA (copies × 10^6^/mL)	NA	2.72 (0.3−11)	1.13 (0.06−16.5)	0.090 ^c^
HIV-RNA (copies/mL)	NA	NA	<20	NA

Results are expressed as median (range). *p* values were calculated by using the ^a^ Kruskal–Wallis test, the ^b^ χ-Square test, or the ^c^ Mann–Whitney test. ALT: Alanine aminotransferase; AST: Aspartate aminotransferase; GGT: gamma glutamiltranspepsidasi; PLT: Platelet; Tot BIL: total bilirubin; NA: not analyzed.

## References

[1] Guidotti L.G., Chisari F.V. (2006). Immunobiology and pathogenesis of viral hepatitis. Annu. Rev. Pathol..

[2] Iredale J.P., Thompson A., Henderson N.C. (2013). Extracellular matrix degradation in liver fibrosis: Biochemistry and regulation. Biochim. Biophys. Acta.

[3] Visse R., Nagase H. (2003). Matrix metalloproteinases and tissue inhibitors of metalloproteinases: Structure, function, and biochemistry. Circ. Res..

[4] Vandooren J., van den Steen P.E., Opdenakker G. (2013). Biochemistry and molecular biology of gelatinase B or matrix metalloproteinase-9 (MMP-9): The next decade. Crit. Rev. Biochem. Mol. Biol..

[5] Arpino V., Brock M., Gill S.E. (2015). The role of TIMPs in regulation of extracellular matrix proteolysis. Matrix Biol..

[6] Lichtinghagen R., Huegel O., Seifert T., Haberkorn C.I., Michels D., Flemming P., Bahr M., Boeker K.H. (2000). Expression of matrix metalloproteinase-2 and -9 and their inhibitors in peripheral blood cells of patients with chronic hepatitis C. Clin. Chem..

[7] Bruno C.M., Valenti M., Bertino G., Ardiri A., Consolo M., Mazzarino C.M., Amoroso A., Neri S. (2009). Altered pattern of circulating matrix metalloproteinases-2,- 9 and tissue inhibitor of metalloproteinase-2 in patients with HCV-related chronic hepatitis. Relationship to histological features. Panminerva Med..

[8] Lichtinghagen R., Bahr M.J., Wehmeier M., Michels D., Haberkorn C.I., Arndt B., Flemming P., Manns M.P., Boeker K.H. (2003). Expression and coordinated regulation of matrix metalloproteinases in chronic hepatitis C and hepatitis C virus-induced liver cirrhosis. Clin. Sci..

[9] Mastroianni C.M., Lichtner M., Mascia C., Zuccalà P., Vullo V. (2014). Molecular mechanisms of liver fibrosis in HIV/HCV coinfection. Int. J. Mol. Sci..

[10] Sulkowski M.S., Thomas D.L. (2003). Hepatitis C in the HIV-infected person. Ann. Intern. Med..

[11] Kim A.Y., Chung R.T. (2009). Coinfection with HIV-1 and HCV—A one-two punch. Gastroenterology.

[12] Hajarizadeh B., Grebely J., Dore G.J. (2013). Epidemiology and natural history of HCV infection. Nat. Rev. Gastroenterol. Hepatol..

[13] Brau N., Salvatore M., Rios-Bedoya C.F., Fernandez-Carbia A., Paronetto F., Rodriguez-Orengo J.F., Rodríguez-Torres M. (2006). Slower fibrosis progression in HIV/HCVcoinfected patients with successful HIV suppression using antiretroviral therapy. J. Hepatol..

[14] Mastroianni C.M., Liuzzi G.M., D’Ettorre G., Lichtner M., Forcina G., di Campli N.F., Riccio P., Vullo V. (2002). Matrix metalloproteinase-9 and tissue inhibitors of matrix metalloproteinase-1 in plasma of patients coinfected with HCV and HIV. HIV Clin. Trials.

[15] Mastroianni C.M., Liuzzi G.M. (2007). Matrix metalloproteinase dysregulation in HIV infection: Implications for therapeutic strategies. Trends Mol. Med..

[16] Fried M.W., Peter J., Hoots K., Gaglio P.J., Talbut D., Davis P.C., Key N.S., White G.C., Lindblad L., Rickles F.R. (2002). Hepatitis C in adults and adolescents with hemophilia: A randomized, controlled trial of interferon alfa-2b and ribavirin. Hepatology.

[17] Bacon B.R., Gordon S.C., Lawitz E., Marcellin P., Vierling J.M., Zeuzem S., Poordad F., Goodman Z.D., Sings H.L., Boparai N. (2011). Boceprevir for previously treated chronic HCV genotype 1 infection. N. Engl. J. Med..

[18] Leroy V., Monier F., Bottari S., Trocme C., Sturm N., Hilleret M.N., Morel F., Zarski J.P. (2004). Circulating matrix metalloproteinases 1, 2, 9 and their inhibitors TIMP-1 and TIMP-2 as serum markers of liver fibrosis in patients with chronic hepatitis C: Comparison with PIIINP and hyaluronic acid. Am. J. Gastroenterol..

[19] Reif S., Somech R., Brazovski E., Reich R., Belson A., Konikoff F.M., Kessler A. (2005). Matrix metalloproteinases 2 and 9 are markers of inflammation but not of the degree of fibrosis in chronic hepatitis C. Digestion.

[20] Capone F., Guerriero E., Sorice A., Maio P., Colonna G., Castello G., Costantini S. (2002). Characterization of metalloproteinases, oxidative status and inflammation levels in the different stages of fibrosis in HCV patients. Clin. Biochem..

[21] Hemmann S., Graf J., Roderfeld M., Roeb E. (2007). Expression of MMPs and TIMPs in liver fibrosis—A systematic review with special emphasis on anti-fibrotic strategies. J. Hepatol..

[22] Duarte S., John Baber J., Fujii T., Coito A.J. (2015). Matrix metalloproteinases in liver injury, repair and fibrosis. Matrix Biol..

[23] Liuzzi G.M., Mastroianni C.M., Latronico T., Mengoni F., Fasano A., Lichtner M., Vullo V., Riccio P. (2004). Anti-HIV drugs decrease the expression of matrix metalloproteinases in astrocytes and microglia. Brain.

[24] Latronico T., Liuzzi G.M., Riccio P., Lichtner M., Mengoni F., D’Agostino C., Vullo V., Mastroianni C.M. (2007). Antiretroviral therapy inhibits matrix metalloproteinase-9 from blood mononuclear cells of HIV-infected patients. AIDS.

[25] Liu T., Wang X., Karsdal M.A., Leeming D.J., Genovese F. (2012). Molecular serum markers of liver fibrosis. Biomark. Insights.

[26] Larrousse M., Laguno M., Segarra M., de Lazzari E., Martinez E., Blanco J.L., León A., Deulofeu R., Miquel R., Milinkovic A. (2007). Noninvasive diagnosis of hepatic fibrosis in HIV/HCV-coinfected patients. J. Acquir. Immune Defic. Syndr..

[27] Lin W., Weinberg E.M., Chung R.T. (2013). Pathogenesis of accelerated fibrosis in HIV/HCV co-infection. J. Infect. Dis..

[28] Deng L., Gui X., Zhang Y., Gao S.C., Yang R.R. (2009). Impact of human immunodeficiency virus infection on the course of hepatitis C virus infection: A meta-analysis. World J. Gastroenterol..

[29] Monga H.K., Rodriguez-Barradas M.C., Breaux K., Khattak K., Troisi C.L., Velez M., Yoffe B. (2001). Hepatitis C virus infection-related morbidity and mortality among patients with human immunodeficiency virus infection. Clin. Infect. Dis..

[30] Martinez-Sierra C., Arizcorreta A., Díaz F., Roldán R., Martín-Herrera L., Pérez-Guzmán E., Girón-González J.A. (2003). Progression of chronic hepatitis C to liver fibrosis and cirrhosis in patients coinfected with hepatitis C virus and human immunodeficiency virus. Clin. Infect. Dis..

[31] Thein H., Yi Q., Dore G.J., Krahn M.D. (2008). Natural history of hepatitis C virus infection in HIV-infected individuals and the impact of HIV in the era of highly active antiretroviral therapy: A meta-analysis. AIDS.

[32] Qurishi N., Kreuzberg C., Luchters G., Effenberger W., Kupfer B., Sauerbruch T., Rockstroh J.K., Spengler U. (2003). Effect of antiretroviral therapy on liver-related mortality in patients with HIV and hepatitis C virus coinfection. Lancet.

[33] Li Y., Xie J., Han Y., Wang H., Lv W., Guo F., Qiu Z., Li Y., Du S., Song X. (2016). Combination antiretroviral therapy is associated with reduction in liver fibrosis scores in HIV-1-infected subjects. Medicine.

